# Cardiovascular magnetic resonance (CMR) and positron emission tomography (PET) imaging in the diagnosis and follow-up of patients with acute myocarditis and chronic inflammatory cardiomyopathy: A review paper with practical recommendations on behalf of the European Society of Cardiovascular Radiology (ESCR)

**DOI:** 10.1007/s10554-023-02927-6

**Published:** 2023-09-08

**Authors:** Federico Caobelli, Jordi Broncano Cabrero, Nicola Galea, Philip Haaf, Christian Loewe, Julian A. Luetkens, Giuseppe Muscogiuri, Marco Francone

**Affiliations:** 1 Department of Nuclear Medicine, Inselspital, Bern University Hospital and University of Bern, Freiburgstrasse 18, Bern, 3000 Switzerland; 2 Cardiothoracic Imaging Unit, Hospital San Juan de Dios. HT médica, Córdoba, Spain; 3 Department of Radiological, Oncological and Pathological Sciences, Sapienza University of Rome, Viale Regina Elena 324, Rome, 00161 Italy; 4 Department of Cardiology, Cardiovascular Research Institute Basel (CRIB), University Hospital Basel, and University of Basel, Petersgraben 4, Basel, CH-4031 Switzerland; 5 Division of Cardiovascular and Interventional Radiology, Department of Bioimaging and Image-Guided Therapy, Medical University Vienna, Spitalgasse 9, Vienna, A-1090 Austria; 6 Department of Diagnostic and Interventional Radiology, University Hospital Bonn, Venusberg-Campus 1, 53127 Bonn, Germany; 7 Department of Radiology, ASST Papa Giovanni XXIII Hospital, Bergamo, Italy; 8 Department of Biomedical Sciences, Humanitas University, Via Rita Levi Montalcini 4, Pieve Emanuele, Milan, 20072 Italy; 9 IRCCS Humanitas Research Hospital, Via Manzoni 56, Rozzano, Milan, 20089 Italy

**Keywords:** Myocarditis, Chronic inflammatory cardiac disease, Cardiac magnetic resonance, Cardiac positron emission tomography, Noninvasive diagnosis

## Abstract

Advanced cardiac imaging techniques such as cardiovascular magnetic resonance (CMR) and positron emission tomography (PET) are widely used in clinical practice in patients with acute myocarditis and chronic inflammatory cardiomyopathies (I-CMP). We aimed to provide a review article with practical recommendations from the European Society of Cardiovascular Radiology (ESCR), in order to guide physicians in the use and interpretation of CMR and PET in clinical practice both for acute myocarditis and follow-up in chronic forms of I-CMP.

## Introduction

**Fig. 1:**
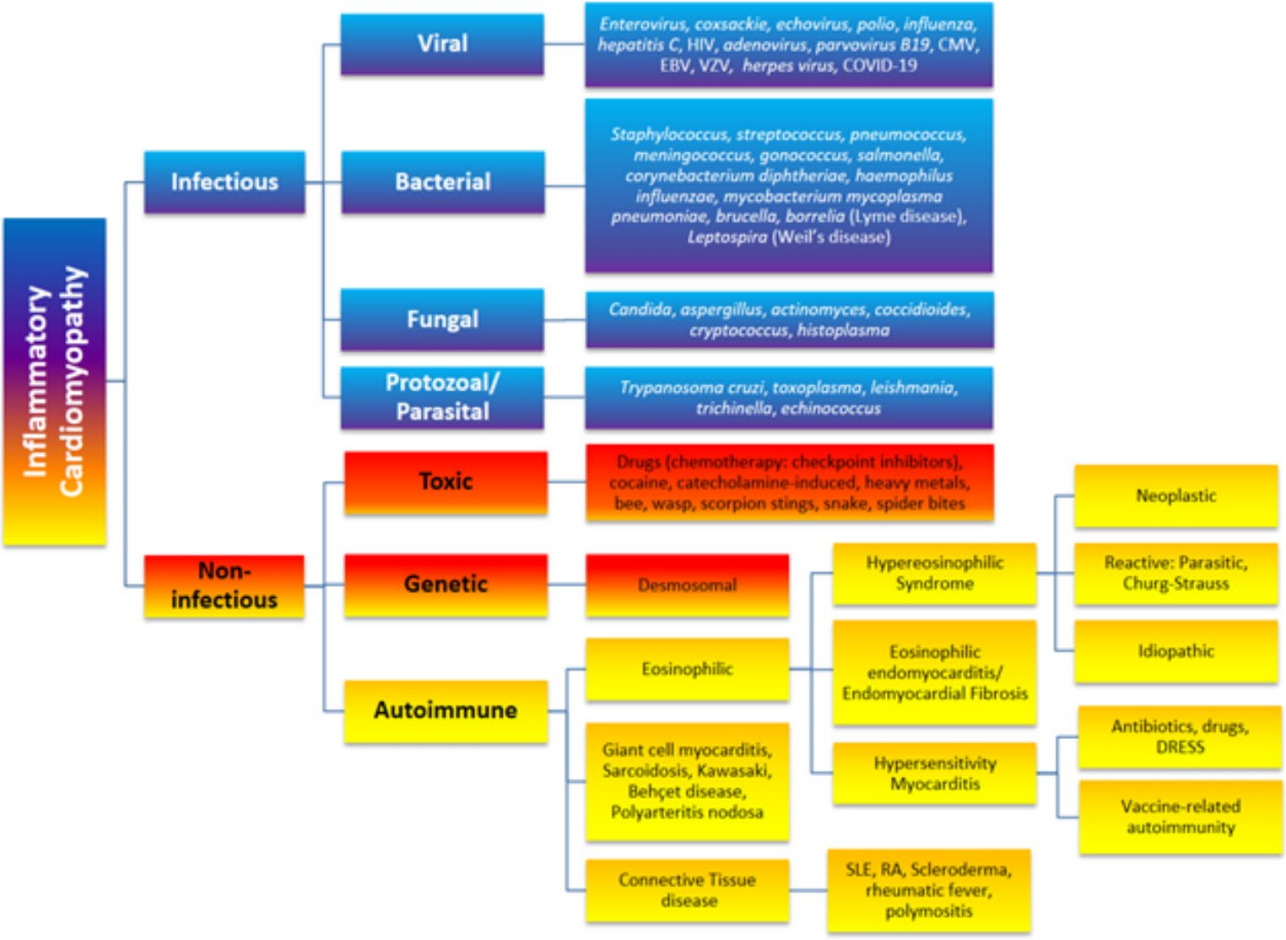
Aetiology of inflammatory cardiomyopathies. HIV = Human Immunodeficiency Virus; CMV = Cytomegalovirus; EBV = Epstein-Barr Virus; VZV = Varicella Zoster Virus; DRESS = Drug Reaction with Eosinophilia and Systemic Symptoms; SLE = Systemic Lupus Erythematosus; RA = Rheumatoid Arthritis. NB: The term “Autoimmune” embraces auto-inflammatory and immune-mediated inflammatory diseases (IMID). Connective tissue disease is also known as autoimmune rheumatic disease. Churg-Strauss syndrome is also known as eosinophilic granulomatosis with polyangiitis (EGPA). Adapted from [2]

Advanced cardiac imaging techniques such as cardiovascular magnetic resonance (CMR) and positron emission tomography (PET) are widely used in clinical practice in patients with acute myocarditis and chronic inflammatory cardiomyopathies (I-CMP). I-CMPs are characterised by inflammatory cell infiltration into the myocardium in association with cardiac dysfunction, ventricular remodelling and have both infectious and non-infectious aetiology (Fig. 1) [1]. Virally mediated cardiac injury is the most common cause of acute myocarditis. A complex interplay of genetic, autoimmune, and environmental factors contributes to the highly variable risk of deteriorating cardiac function, acute heart failure, and arrhythmia as well as chronic dilated cardiomyopathy and its sequelae [2]. The reason why some patients with myocardial inflammation recover without residual myocardial injury whereas others develop dilated cardiomyopathy remains unclear.

CMR and PET have become key tools to non-invasively diagnose acute myocarditis and I-CMPs, visualize and understand pathophysiological mechanisms and to better identify patients at risk of developing heart failure and dilated cardiomyopathy. They have largely reduced the need for endomyocardial biopsy (EMB) in hemodynamically stable patients given its limitations such as invasiveness, availability, costs and sampling error due to the predominantly *subepicardial* and *mid-myocardial* wall involvement in acute myocarditis and I-CMP [1, 3].

We aimed to provide a review article with practical recommendations to guide physicians in the use, and interpretation of CMR and PET in clinical practice both for acute myocarditis and follow-up in chronic forms of I-CMP.

## Diagnosis of acute myocarditis and inflammatory cardiomyopathy

The diagnosis of acute myocarditis and I-CMP is based on a combination of clinical history, electrocardiogram, blood tests, cardiac imaging, and where necessary, EMB.

### Endomyocardial biopsy

EMB should be considered in all cases with presumed giant cell myocarditis and fulminant myocarditis (severe heart failure/cardiogenic shock), malignant ventricular arrhythmia or high-grade atrioventricular block (II° or III°). Given the limited diagnostic accuracy of CMR in identifying the specific aetiology of myocardial inflammation, EMB may also be indicated in patients with a presumed cardiac sarcoidosis, eosinophilic myocarditis or systemic inflammatory disease for which there are specific treatment options available apart from general heart failure treatment [4–8]. Of note, myocardial inflammation often involves the (sub-)epicardial and mid-myocardial walls and the left ventricle whereas EMB preferentially is done from samples of the (sub-)endocardial layers of the right ventricle. EMB may therefore lead to sampling errors, the sensitivity of EMB has been reported to be higher for giant cell myocarditis (80–93%) than for sarcoidosis (25%) and lymphocytic myocarditis (35%) [9, 10].

### CMR

Although EMB is still the reference standard to prove a diagnosis of myocarditis and its etiology, there has been a notable shift for the diagnosis of myocarditis over the last decades towards a non-invasive approach. CMR offers non-invasive imaging that can accurately assess myocardial inflammation and is now considered the first-line modality to confirm suspected inflammatory myocardial disease. CMR is basically recommended [4] to *confirm* diagnosis in clinically suspected acute myocarditis (onset of symptoms < 30 days, mostly infarct-like presentation) or [5] to *evaluate* the presence of chronic myocarditis or chronic inflammatory cardiomyopathy (I-CMP) in patients with persistent cardiac symptoms (onset of symptoms > 30 days, persistent troponinaemia, mostly presentation with heart-failure-like symptoms or unexplained arrhythmias) [4, 5].

**Fig. 2:**
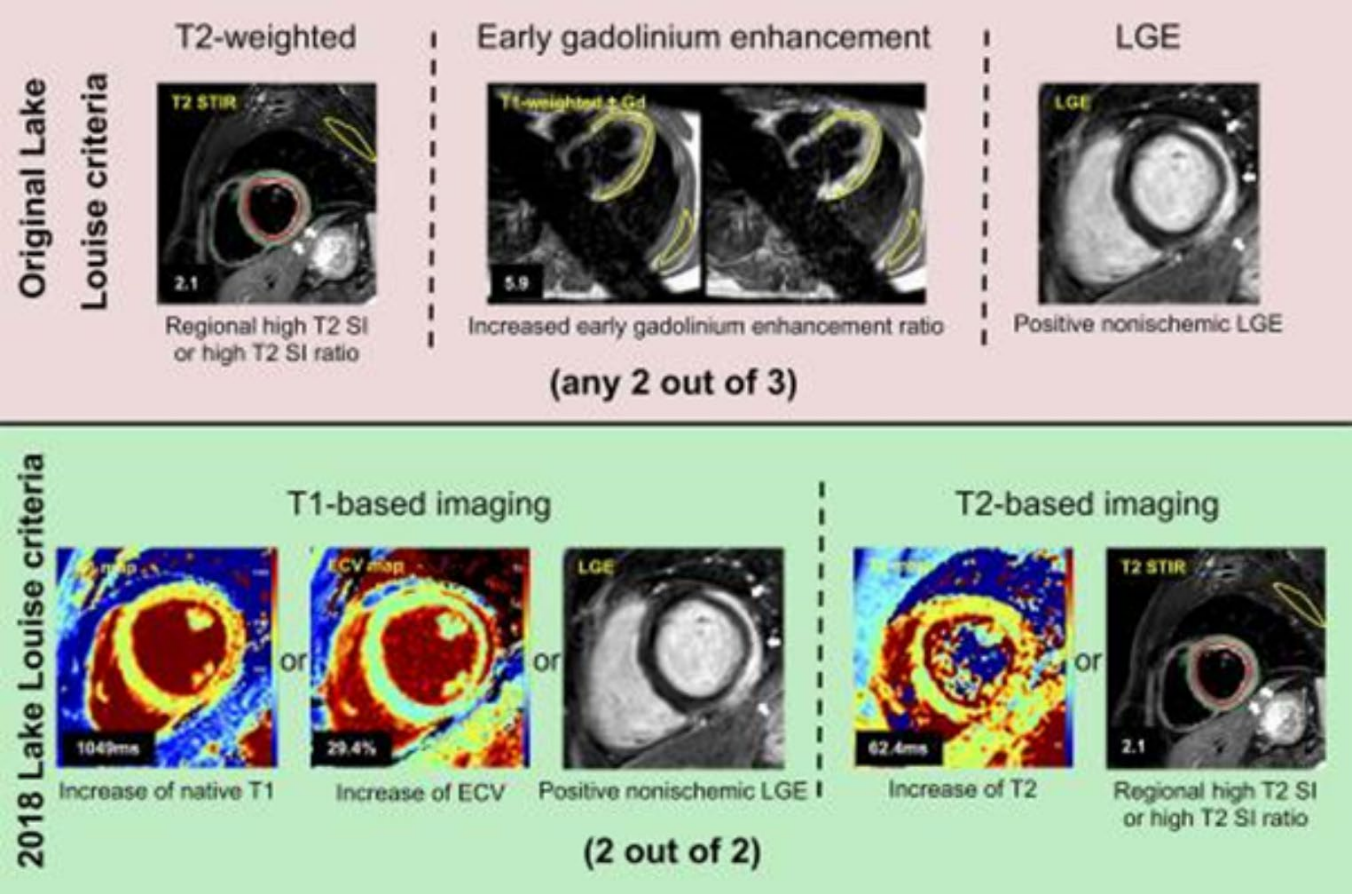
Original and 2018 Lake Louise criteria (LLC) in a 24-year-old man with acute myocarditis. Original LLC consisted of three main criteria: regional high T2 signal intensities on T2-weighted images (white arrows) or increased global T2 signal intensity ratio, increased early gadolinium enhancement ratio on T1-weighted images, and areas with high signal intensities in nonischemic distribution pattern on late gadolinium enhancement (LGE) images (white arrows). 2018 LLC consist of two main criteria (T1-based criterion and T2-based criterion). T1-based criterion is considered to be positive if increase of native T1 relaxation times, increase of extracellular volume (ECV), or positive LGE (white arrows) exist. T2-based criterion is positive in cases of increased T2 relaxation times or in cases with regional high T2 signal intensities on T2-weighted images (white arrows) or increased global T2 signal intensity ratio. Gd = gadolinium, SI = signal intensity, STIR = short tau inversion recovery.Reprinted from [12]. No changes were made

CMR is recommended in clinically stable patients with acute symptoms to confirm clinical suspicion of acute myocarditis by demonstrating inflammatory necrosis and myocardial oedema [1, 4]. The diagnostic accuracy of CMR following Lake Luis Criteria (LLC) is high for acute infarct-like presentations (a diagnostic accuracy up to 90% can be achieved, Fig. 2) [11, 12]. However, the sensitivity of CMR in biopsy-proven acute myocarditis depends on the type of clinical presentation and is lower for chronic cardiomyopathic, and very low for arrhythmic patterns (sensitivities: 40-57%) [11]. In most stable patients with presumed myocarditis, CMR will be sufficient for confirming diagnosis. In high-risk patients with cardiogenic shock or fulminant clinical course, EMB should be first and foremost performed [1, 4]. Nevertheless, in experienced medical centers with interdisciplinary teams of radiologists, anesthesiologists, and cardiologists, CMR can be performed even in intubated intensive care patients (if mechanical circulatory support is not required) to guide subsequent EMB [13]. Myocardial mapping techniques have further improved diagnostic accuracies over the last years, especially for the detection of diffuse myocardial oedema and inflammatory processes [12, 14, 15]. Moreover, CMR offers prognostic value by the assessment of disease activity and severity including ventricular remodeling and function, myocardial inflammation (oedema and necrosis), and myocardial fibrosis [16].

In the work-up of patients with unexplained heart-failure symptoms or ventricular arrhythmias, CMR is recommended to exclude chronic inflammatory myocardial disease [4, 5]. CMR can help to differentiate between ischaemic or non-ischaemic myocardial disease by visualization of the pattern of myocardial scarring (I.e., subendocardial scarring with matching a coronary artery territory, as a sign for ischaemic injury) and fibrosis and work as a gatekeeper for potential EMB. Due to their higher sensitivity for the detection of diffuse myocardial edema and fibrosis, the application of T1 and T2 mapping can be of particular value in patients with chronic myocarditis or chronic I-CMP [17]. High-sensitivity cardiac troponin (hs-cTn) assays are very sensitive but non-specific markers of myocyte injury and almost invariably elevated in patients with acute or ongoing myocardial inflammation [18] Myocardial inflammation may rarely occur with normal hs-cTn levels e.g. in patients treated with immune checkpoint inhibitors (probably only the presence of myocardial oedema without myocyte injury) [19]. Therefore, hs-cTn assays may be used to exclude ongoing myocardial inflammation in the vast majority of patients [8]. EMB should be considered to exclude low-grade myocardial inflammation in patients with negative CMR result but refractory cardiac symptoms and persistent suspicion of chronic inflammation.

Furthermore, CMR can be indicated to evaluate adverse effects of different treatments, e.g. traditional and new anticancer therapies, in patients with suspected cardiotoxicity including immune checkpoint inhibitor myocarditis [5, 20–22].

### PET

Positron emission tomography (PET) with 2-deoxy-2-[18 F]-fluoro-D-glucose (^18^ F-FDG) has gained interest in the last years, owing to its capability to reveal focal or diffuse patterns of inflammation as seen in myocarditis.

Glucose is a normal metabolic substrate of myocardium, which is normally used in clinical practice for exploring myocardial viability [23]. Due to the physiologic ^18^ F-FDG uptake within the myocardium, a specific patient’s preparation is needed to assess the presence of inflammatory foci. Therefore, long fasting, low-carbohydrates and high-fat meal and/or fractionated/unfractionated heparin administration before ^18^ F-FDG injection are commonly used to suppress physiological radiotracer uptake and increase PET specificity [24–26].

To date, ^18^ F-FDG PET has been suggested for the noninvasive diagnosis of myocarditis, to guide EMB, and for monitoring treatment response. However, as large clinical trials are lacking so far, the use of ^18^ F-FDG PET as standalone modality in the diagnostic workup cannot be recommended, possibly with the exception of cardiac sarcoidosis [2].

Recently, new hybrid PET-CMR scanners became available in clinical practice, and they represent an attractive imaging modality for the evaluation of myocarditis and I-CMP. In fact, PET-CMR has the advantage of allowing simultaneous acquisition of both CMR and PET combining the morphological and ventricular functional data, tissue characterization, and metabolic information in the same examination [27]. However, due to higher costs and limited availability compared to standalone modalities, the use hybrid PET/MR imaging is not widespread yet.

## Imaging findings

### CMR

**Fig. 3:**
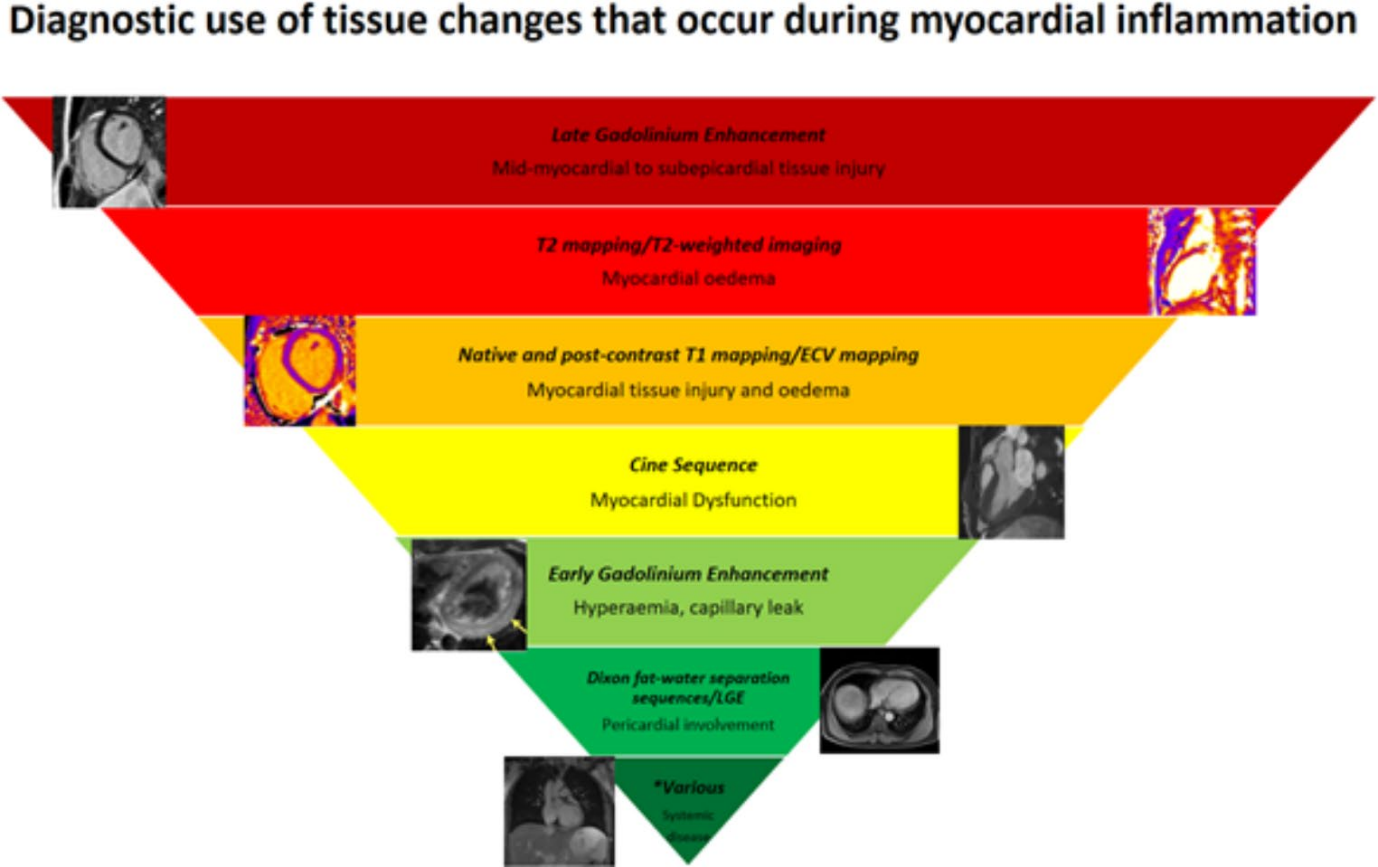
Weighting of CMR imaging findings in the diagnosis of inflammatory cardiomyopathy. *Various  includes CMR sequences such as post-contrast fat suppressed techniques and localizers

CMR is ideal to illustrate most of the historical hallmarks of inflammation: 1: rubor/calor (Oedema sequences), 2: dolor (patient history), 3: tumor (transient elevated myocardial mass/“hypertroph” due to oedema), 4: function laesa (Ejection fraction, regional wall motion abnormalities). Furthermore, it enables a multiparametric assessment that combines the evaluation of myocardial tissue abnormalities, the impairment of the contractile function and the pericardial involvement. The presence of myocardial oedema, hyperaemia, necrosis and/or fibrosis represents the typical features of inflammatory damage and allow to assess the extent and degree of activity of the myocardial injury (Fig. 3).

#### Myocardial oedema

Myocardial oedema, defined as an increase in water content in myocardial tissue due to the expansion of the interstitial fluid, can be depicted by T2 weighted images (double or triple inversion recovery with blood and fat suppression) [28] as implemented signal intensity (SI) areas as compared to the not injured myocardium [29]. The distribution of these tissue abnormalities is mostly confined to the mid-myocardium and subepicardium but may also occur transmurally or subendocardially. Myocardial oedema may occur globally but more frequently in a regional pattern and in association with occurrence of acute late gadolinium enhancement. In contrast to ischaemia-associated myocardial damage, oedema in myocarditis typically does not occur in a coronary artery pattern [30].

**Fig. 4:**
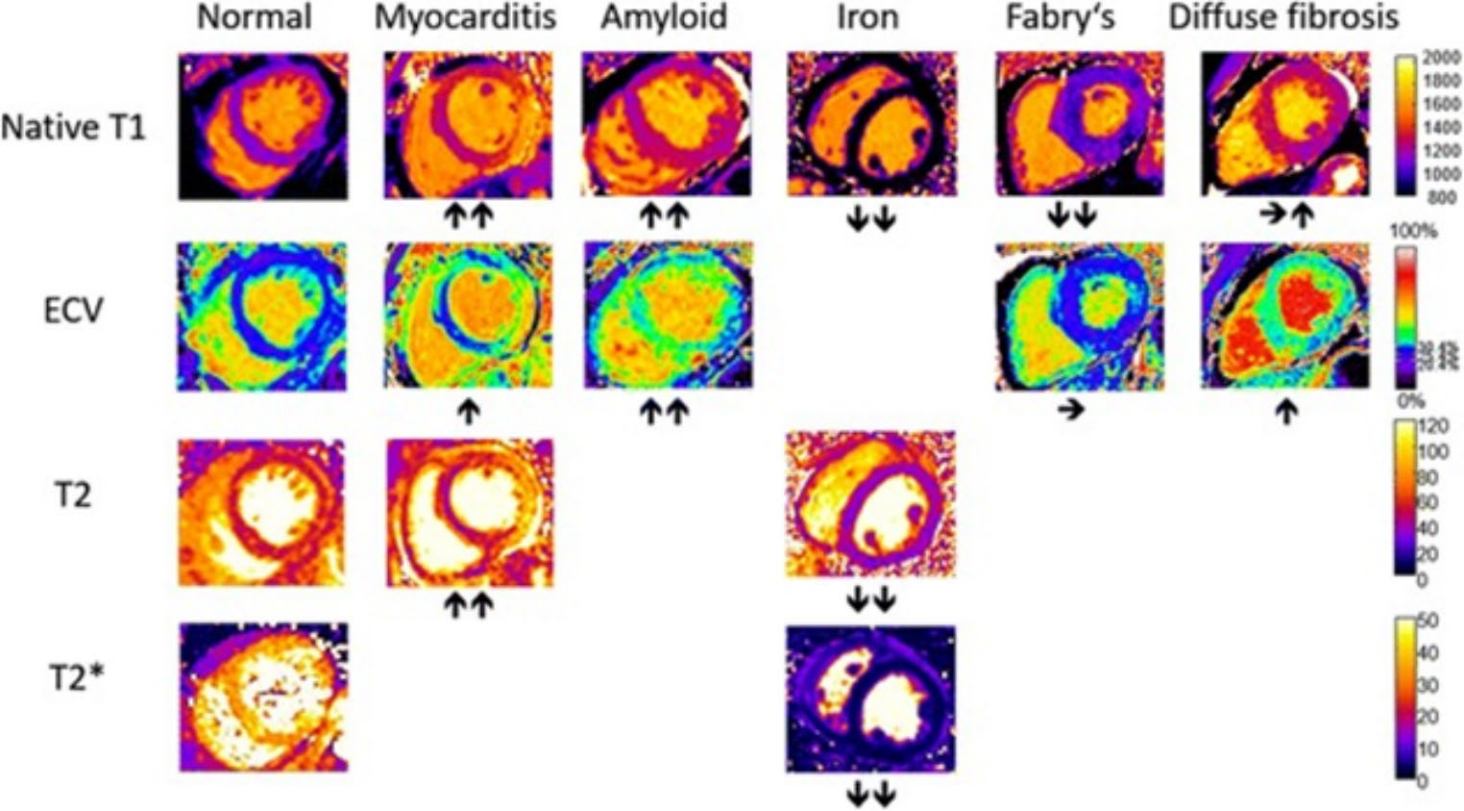
Typical appearance of T1, T2, T2*, and ECV maps in healthy subjects and in patients with myocardial disease. Arrows denote relative change in respective parametric maps. Reprinted under the terms of the Creative Commons Attribution 4.0 International License from [33]. No changes were made

The intrinsic limitations in evaluating the myocardial edema when the T2 SI is diffusely increased can be overcome by the semi-quantitative analysis based on the normalization of myocardial SI to that of the skeletal muscle. A myocardial-to-skeletal muscle T2 signal ratio > 2 may be considered consistent for the presence of edema [31]. New approaches rely on relaxometric sequences: T2 mapping is highly specific for edema detection (area under the curve (AUC) 0.85–0.91 [32]) since those sequences are based on direct calculation of T2 relaxation times [33], and therefore highly specific for the acute setting of the disease [14]. T1 mapping sequences can also reveal the presence of edema (AUC 0.94–0.95 [34, 35]), but with lower specificity than T2 mapping, due to different mechanisms associated to an increase in T1 values [14, 33, 36]. T1 and T2 mapping sequences are able to detect edema even if diffuse and not easily evaluated with conventional sequences mainly based on the visual assessment of the disease (Fig. 4) [33].

#### Hyperaemia and capillary leak

Hyperaemia reflects the increased permeability of the vessels associated to the inflammatory response. The detection of this phenomenon results to be the most difficult and challenging for CMR [37]. According to the old Lake Louise criteria (LLC), hyperemia and capillary leakage were evidenced by increased SI with T1-weighted spin echo (T1-SE) sequences acquired early after contrast media administration compared to pre-contrast T1 - SE ones [31]. Historically, the semi-quantitative analysis defined the presence of hyperemia when SI ratio is > 4 as compared to the skeletal muscle or when the absolute myocardial enhancement is > 45% [31]. However, by definition, hyperemia is a dynamic process theoretically influenced by time-variations of tissue enhancement and therefore the technique for its evaluation, based on static images obtained with long acquisition times, suffers from low robustness and accuracy. Consequently, this criterion was excluded from the revised LLC, based on the low AUC demonstrated in several studies, ranging from 0.62 to 0.93 (Fig. 3) [14]. A promising prospective is offered by the measurement of early T1 shortening, measured by the percentage of T1 value reduction on T1 maps acquired early after administration of contrast medium (sensitivity/specificity of 93%/95% for early T1 shortening ≧ 70%) [38].

#### Necrosis and fibrosis

Necrosis, and subsequent fibrosis, represents the irreversible step of myocardial injury induced by the inflammatory cascade and are both associated with alteration of the permeability of the sarcolemma resulting in a myocardial accumulation of gadolinium [14].

LGE imaging has proven to be a valid tool for the detection of such damage, showing a high specificity [39] through the identification of common patterns of the regional distribution of non-ischemic myocardial injury [40]. Myocarditis-associated LGE lesions usually involve subepicardium and mid-wall and tends to favor basal to mid-inferolateral wall in a non-coronary artery pattern [41]. Nevertheless, severe inflammation can rarely lead to the extension of LGE area to the entire myocardial wall [31]. The solitary use of LGE for diagnosing myocarditis, however, is not recommended, due to its low specificity for acute inflammation. In this regard, Radunski et al. demonstrated a better diagnostic accuracy of T1 and ECV mapping, which increase the sensibility of CMR in the detection and quantification of diffuse myocardial fibrosis compared to LGE images [42].

Furthermore, the LGE areas may persist even in the chronic phase, when the inflammatory activity subsides, with possible shrinkage of the areas of enhancement, in relation to scar remodeling phenomena. It should be noted that in the acute phase it is often impossible to say whether LGE is a sign of focal (irreversible/chronic) fibrosis or oedema. In this situation, FDG PET may aid in the diagnostic definition.

#### Ventricular geometry and functional abnormalities

Myocardial inflammation may lead to regional or global left ventricle (LV) and right ventricle (RV) dysfunction (function laesa) [31]. However, wall motion abnormalities in myocarditis are often focal and can be compensated by a hypercontractility of the surrounding myocardium, which can mask the dysfunction [14]. Even with significant tissue injury, there may be remarkably little impact on cardiac contractility, as the endocardial myocytes, which tend to be prime movers in normal ventricular function are often relatively spared in acute myocarditis [8]. In this regard, myocardial strain can be helpful in detecting subtle wall motion abnormalities, resulting in an increased sensitivity of CMR [43].

However, alterations in regional contractility or global systolic function can underlie multiple pathological conditions, not necessarily related to a direct myocardial insult. Therefore, functional abnormalities should be considered as an ancillary criterion for the diagnosis of myocarditis [14].

#### Pericardial involvement

Pericarditis and myocarditis often coexist, due to the common etiologic agents and overlapping pathophysiological mechanisms. Although pericardial effusion is a common finding in patients with myocarditis, its presence alone is not sufficient for the diagnosis of pericarditis or myo-pericarditis [44].

Acute pericardial inflammation may be depicted by CMR as thickening of pericardial layers in high-resolution fast spin echo (FSE) T1 images, hyperintensity of the pericardial layers on T2-weighted, or pericardial enhancement on ECG-gated Dixon fat-water separation sequences or LGE images [45].

### PET

Typically, areas of active inflammation present with increased ^18^ F-FDG uptake, which could be focal, diffuse, or focal on diffuse depending on the underlying nature of the disease [46]. Such areas of increased ^18^ F-FDG uptake may show a resolution after treatment, thus holding the potential for monitoring treatment response [47].

A retrospective study featuring 29 symptomatic patients showed that there is an excellent correlation between ^18^ F-FDG PET/CT and EMB from left ventricular posterior wall. Of note, the authors suggested that the best timing of imaging is within 14 days after the onset of clinical symptoms [46]. Moreover, another paper by Perel-Winkler et al. using ^18^ F-FDG-PET/CT in patients with systemic lupus erythematosus [48] showed diffuse myocardial ^18^ F-FDG uptake in those patients with chest pain, dyspnea and/or impairment of left ventricular ejection fraction (LVEF). Similar results were reported by Besenyi et al. [49], wherein patients with systemic sclerosis showed both visually and semi-quantitatively higher myocardial ^18^ F-FDG uptake compared to healthy subjects. Hence, there is a strong rationale to suggest that the presence of areas of increased myocardial ^18^ F-FDG uptake in symptomatic patients is highly suggestive for active inflammation, as it can be seen in myocarditis.

**Fig. 5:**
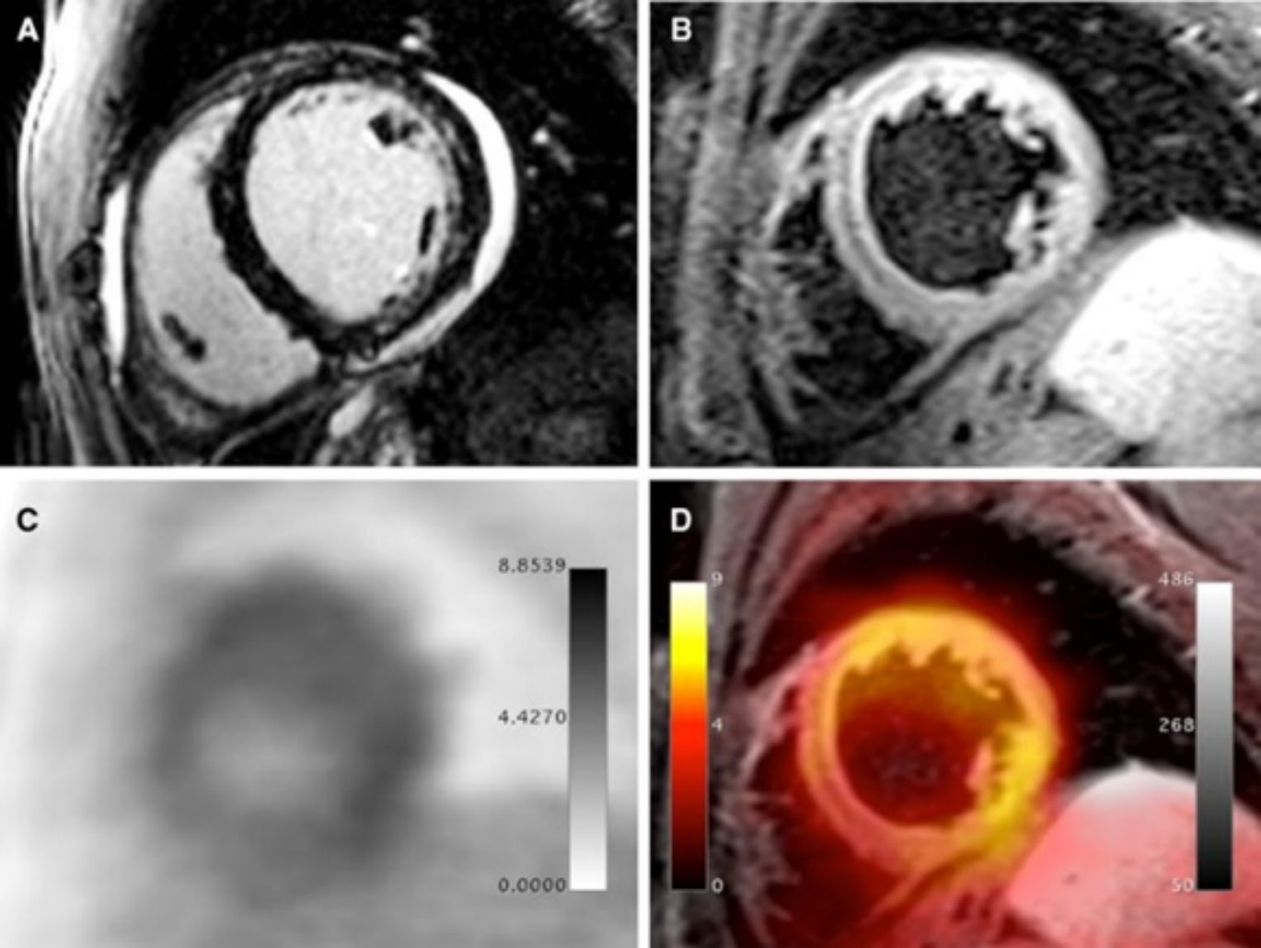
PET/MRI examination in a 32-year-old male patient presenting with dyspnea, mild ventricular dysfunction (51% LFEV), and a history of recent systemic viral disease. **A** shows patchy intramyocardial late gadolinium enhancement in the lateral and inferior wall as well as pericardial effusion. **B** shows significantly increased T2 signal in the lateral wall representing myocardial edema. **C** (PET) and **D** (fusion between T2-weighted MR image and PET) show diffusely increased FDG uptake in the lateral, anterolateral, and inferolateral wall. Histopathological assessment after endomyocardial biopsy showed acute myocarditis with lymphocytic infiltration and moderate myocyte apoptosis. The patient demonstrated elevated levels of C-reactive protein (4.1 mg/dl) as well as elevated myocardiocytolysis serum markers (Troponin-I: 0.42 ng/ml). PCR and immunohistochemical analysis did not detect specific infectious agents such as viruses, bacteria, or fungi. Reprinted with permission of Springer from [50]

**Fig. 6:**
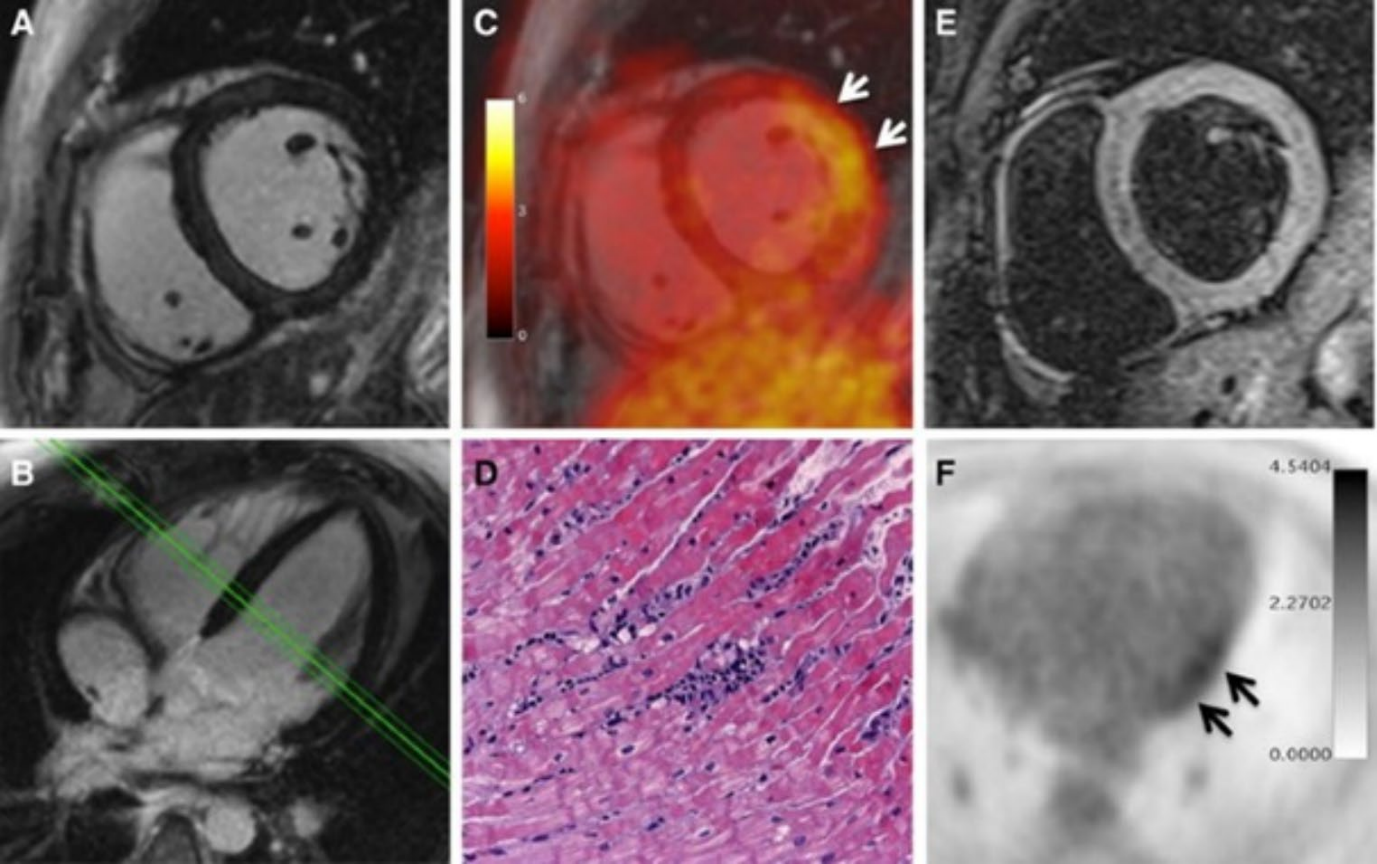
PET/MRI examination in a 30-year-old male patient presenting with chest pain, dyspnea, palpitations, severely limited exercise capacity, mild ventricular dysfunction (54% LVEF), and mild ECG abnormalities (T-wave flattening in II, III, aVF, and V6). The patient demonstrated neither elevated levels of C-reactive protein nor elevated myocardiocytolysis serum markers. LGE images **(A**, **B)** did not reveal any signs of myocardial necrosis. PET images **(C**, **F)** demonstrated focal FDG uptake in the lateral wall. T2-weighted imaging **(E)** showed mild myocardial edema (T2 ratio: 2.0). Diagnosis of borderline myocarditis was confirmed by histopathological assessment after endomyocardial biopsy demonstrating sparse inflammatory infiltrates but no myocardial necrosis **(D)**. PCR and immunohistochemical analysis did not detect specific infectious agents such as viruses, bacteria, or fungi. Reprinted with permission of Springer from [50]

Using a hybrid PET/MR approach, images normally show focal or diffuse ^18^ F-FDG uptake, corresponding to MR alterations (Fig. 5). In a prospective study, a good agreement between the two techniques and feasibility of hybrid imaging has been demonstrated [50]. Of note, preliminary data also show potential incremental role of PET/MR over CMR alone. In fact, LGE may not detect myocardial damage if scattered, and mild borderline myocarditis can be often challenging to reveal with LGE due to the absence of relevant myocardial necrosis [33]. Hence, in selected patients the ^18^ F-FDG PET component may increase the sensitivity of CMR by providing metabolic information (Fig. 6) [51].

### Specific conditions

Specific conditions are summarized in Table 1.

**Table 1:** Specific Conditions for Inflammatory Cardiomyopathy. For all disorders, T2-weighted Images, T2-mapping values and T1-mapping values are abnormal. Further specific findings are listed hereunder for the relevant etiologies

*Infectious Etiology*	
	CMR
Viral myocarditis	• LGE lateral wall or septum (subepicardial or mid-wall) [52, 53] • Low/ normal LVEF
Bacterial myocarditis	• LGE lateral wall (subepicardial or intramural) [54, 55]
Parasitic Myocarditis	• LGE lateral wall (patchy in toxoplasma gondii [56] and midwall, subepicardial, subendocardial or transmural in patients with Trypanosoma cruzi with a prevalence for inferolateral basal and apex) [57, 58]
*Non-Infectious Etiology*	
Eosinophilic myocarditis	• Diffuse subendocardial LGE [59, 60] • Possible association with ventricular thrombus [59]
Systemic Lupus Erythematosus	• Subendocardial, intramyocardial, subepicardial LGE [61, 62]
Systemic Sclerosis	• Prevalent subepicardial, midwall LGE of the septum [63]
Polymyositis	• Subepicardial lateral and inferior wall [64]
Giant Cells Myocarditis	• Subendocardial, and subepicardial diffuse LGE with involvement of both ventricles [65]
Kawasaki Syndrome	• Subendocardial LGE [66]
Sarcoidosis	• Subendocardial, subepicardial, midwall LGE [67]
Chemotherapy	• Subepicardial or mid-wall LGE [68]
Post-Vaccination	• Subepicardial or mid-wall LGE [69, 70]

LGE: late gadolinium enhancement

### CMR follow-up in myocarditis

Most patients (up to 84% in some series) with acute myocarditis have a benign course with full recovery of ventricular function and resolution of myocardial oedema without sequelae (healed myocarditis) [16, 71, 72]. Occasionally, acute myocarditis may induce significant morbidity and mortality, especially in severe forms presenting with ventricular functional impairment [73]. A 5 - year mortality rate of almost 20% in severe forms of acute myocarditis and up to 10% of sudden cardiac death in young adults has been described [74, 75]. Persistent inflammation, often subclinical, with an autoimmune substrate, may lead to dilated cardiomyopathy [71]. Up to 30% of biopsy–proven myocarditis can progress to dilated cardiomyopathy with an associated poor prognosis [1]. Known predictors of poor outcome include viral infection or evidence of immunohistological signs of inflammation on EMB, poor New York Heart Association (NYHA) functional class, impaired LV function or presence/extent of LGE [76].

Most patients with acute myocarditis have a good short-to-midterm clinical evolution with complete resolution of myocardial edema, improvement of LVEF, and reduction of left ventricular mass index (LVMi), being a marker of global myocardial inflammatory infiltration (Table 2). In the follow up reduction or disappearance of LGE in a follow-up CMR scan might refelct reversible injury. CMR with parametric mapping can effectively distinguish healed from active myocarditis [42, 77–79]. After the acute presentation, T1 native and T2 values decreased progressively in the early follow up period, both representing progressive resolution of the myocardial edema [78]. In fact, several studies have revealed a steady decline on T1 native and ECV values from the acute phase to chronic convalescent phase, but being higher than in controls [42, 72, 78–80]. Higher T1 and T2 AUC (0.947 and 0.931, respectively) have been described for discriminating between acute from healed myocarditis compared to LGE and T2 STIR (0.809 and 0.884, respectively) [77]. Also, ECV was the most robust parameter for differentiating healed myocarditis form healthy controls (AUC: 0.925; ECV > 26%, 85.2% sensitivity and 100% specificity) [77]. Malek et al. showed that patients with persistent myocardial inflammation (up to 28%), usually asymptomatic, had myocardial oedema or LGE on the initial CMR scan [81]. Moreover, LGE extent has been associated with adverse remodeling (increased LVEDV index and LV systolic volume index), lower LVEF and occurrence of major cardiovascular events (MACEs) [41, 79, 82, 83]. Because subclinical persistent ongoing inflammation and LGE can lead to dilated cardiomyopathy, heart failure and ventricular arrythmias, several authors have suggested that a CMR follow up may be adequate in patients with acute myocarditis [16, 81].

**Table 2:** CMR biomarkers and short – to – midterm follow up prognosis

Study	year	N	F/U time	Biomarkers	Results
Li et al. [77]	2020	19	3 months	LGE mass, LVMi, T2R, T2, T1 native, ECV	**LGE mass** and **LVMi** significantly decreased on 3 months f/u. **LGE, T2R, T1 native** and **T2** discriminate acute versus healed myocarditis. **ECV** excellent for distinguish healed myocarditis from controls in 3 months f/u
Malek et al. [81]	2020	18	7 months (6–9 months)	T2R, LGE	**T2R** and **LGE**: Patients with persistent inflammation on CMR f/u had higher T2R on the initial CMR, higher median number of segments with LGE, higher LVEDV and mass. CMR monitoring of LVEF could not discriminate ongoing inflammation during f/u.
Von Knobelsdorff – Brenkenhoff et al. [78]	2017	18	5–10 days, 5 weeks and 6 months	T2R, T2, T1 native, ECV and LGE	**T2R** and **T2**: excellent discrimination of acute versus controls. Gradual decrease over time. **T1 native** and **ECV**: Identify diseased patients on baseline. Mildly elevated on healed myocarditis f/u (interstitial fibrosis). **LGE**: Persisted in the majority of patients as a specific marker of irreversible injury.
Faletti et al. [82]	2017	52	6 months (5–8 months)	LVEF, LVMi, T2R, EGE, LGE	Reduction of **LVMi**, increase of **LVEF**, normalization of the **T2R** and **EGE** was observed in most of patients with positive evolution. **LGE**: Persistence with significant reduction of the percentage of LGE.
Berg et al. [79]	2017	24	3 months	LGE	Clinical findings, cardiac enzymes and inflammatory biomarkers may not be sufficient to risk stratify patients in the f/u. **LGE**: Increase LGE > 20% associates with the occurrence of adverse cardiovascular events (arrythmias, chest pain or dyspnea).
Ammirati et al. [83]	2016	49	4–5 months	LGE	Globally, a significant decrease in **%LGE** was observed in acute myocarditis Patients with LVEF < 55% at presentation, the **%LGE** was stable or increased at f/u. Baseline **%LGE** correlated with adverse remodeling (LVESVi) and LVEF. Adverse remodeling was associated with less **%LGE** reduction at f/u.
Luetkens et al. [88]	2016	69	2–3 weeks, 4–8 weeks, and > 8 weeks	T2R, T2, T1 native, ECV and LGE	**T2R and T2**: Decrease over time. Baseline myocardial edema correlated with increase EF in f/u. **Mapping (T1/T2**): Distinguish active versus convalescent myocarditis. **LGE**: Decrease over time. Marker of irreversible myocardial injury.
Marholdt et al. [31]	2006	71	4–5 months	LGE, LVEDV, LVEF	**LGE**: LGE in the ventricular septum and total amount of LGE was strongest independent CMR predictor of impaired ventricular function and dilatation at f/u. **LVEF and LVEDV**: LVEF and LVEDV at presentation combined to PVB19 infection, coinfection, chest pain or HF at presentation were predictors of LV function and dilatation at f/u.

Several studies evaluated the prognostic value of CMR surveillance in the long – term follow up in patients with acute myocarditis (Table 3). Chopra et al. showed that LVEF was lower in patients with MACEs compared to those free of MACEs (48.9 ± 11.5% vs. 57 ± 8.0%; p < 0.05) [84]. Other authors showed that LVEF constituted the best independent predictor of adverse clinical events, incomplete recovery and lower LVEF at follow up [16, 73, 74, 85]. Larger LVEDV index at the initial CMR was associated with lower LVEF at follow up CMR (85.9 ± 21.7 ml/m2 vs. 71.8 ± 17.1 ml/m2 LVEDV index for reduced and preserved LVEF, respecrively; p = 0.02) [85]. Higher extension of reversible myocardial damage was seen in patients without MACES, being an independent predictor of LVEDV and LVEF improvement at follow up (reverse remodeling) [85–87]. LGE extent, presence of LGE without myocardial edema and septal pattern on LGE were independent predictors of MACEs and hospitalization due to heart failure in the follow up.

**Table 3:** CMR biomarkers and long term follow up prognosis

Study	year	N	F/U time	Biomarkers	Results
Gräni et al. [88]	2019	670	4.7 years	LGE	**LGE**: LGE size and extent was associated with MACE (all-cause death, heart failure decompensation requiring hospital admission, heart transplantation, documented sustained ventricular arrythmia and recurrent acute myocarditis).
Aquaro et al. [16]	2019	187	7 years (6–8 years)	LGE, LVEF	**LVEF** and **LGE** extent at the initial CMR, LGE extent, LGE midwall septal pattern, LGE persistence without edema and LGE increase at the CMR f/u were associated with cardiac events. **LGE** midwall septal pattern and persistence of LGE without edema were independent predictors of cardiac events on multivariate analysis.
Bohnen et al. [71]	2017	48	3 months and 12 months	LGE, T1, T2 and ECV	**LGE/ECV**: Strong discriminator between myocarditis (acute and healed) versus healthy individuals. **Native T1 and T2**: Help to discriminate without contrast media acute versus healed myocarditis in the f/u.
Chopra et al. [84]	2016	88	16–50 months	LVEF, RVEF, LGE	**LVEF and RVEF** was lower in patients with MACEs than without MACEs **LGE extension** was higher in patients with MACEs rather than free of MACEs. **LGE mass** was an independent predictor for MACE occurrence.
Sanguineti et al. [85]	2015	203	18.9 ± 8.2 months	T2, EGE, LVEF, LVEDV	Extension of **T2** damage and **EGE** was greater in patients without MACEs **LVEF**: Lower initial LVEF was an independent predictor of adverse clinical outcome at f/u and lower LVEF at f/u. **LVEDV**: Larger LVEDV at initial presentation was associated with altered LVEF at f/u.
Schumm et al. [73]	2014	405	1591 days	LVEF, LGE	Patients with clinical suspected myocarditis and normal CMR have excellent long-term prognosis. CMR measured **LVEF** constitute the best independent predictor of cardiac mortality. **LGE** and **LVEF** were independent predictors of MACE and hospitalization due to heart failure.
Vermes et al. [86]	2014	37	12 months	T2R, EGE, LGE	Positive LL criteria was associated with lower LVEF and higher LVESV at baseline and lower LVEF at 1-year f/u. Global/regional myocardial edema (**T2R**) was associated with increase in LVEF > 5%. Global / regional myocardial edema (**T2R**) was an independent predictor for improvement of systolic function.
Grün et al. [74]	2012	203	4.7 years	LGE, LVEF	**LGE**: best independent predictor of all-cause mortality and cardiac related mortality. No patient without LGE experienced SCD. **LVEF**: NYHA class followed by LVEF in the f/u were the best independent predictors for incomplete recovery.
Mavrogeni et al. [89]	2011	71	12 months	EGE, LGE	**EGE**: Negative correlation between EGE and LVEF both at initial evaluation and 1-year f/u. **LGE**: Negative correlation between LGE after 1-year f/u and LVEF.
Zagrosek et al. [87]	2009	36	18 ± 10 months	T2R, EGE and LGE	**T2R** and **EGE** decreased at f/u (reversible damage) with improvement of LV functional parameters. **LGE**: Persisted over the entire course of myocarditis (marker of irreversible damage). **T2R** in the acute phase was an independent predictor of the change in LVEDV at f/u.

Although advantages have been described in the literature regarding the value of CMR in tissue characterization and risk stratification in the surveillance of I-CMP, there is currently no consensus on the use and timing of CMR during I-CMP follow up. Follow up CMR in I-CMP may be considered in patients with adverse cardiac remodeling (increased LVEDV or LVSV index), impaired LVEF (< 50%), extensive reversible myocardial damage or abundant LGE (in particular with septal or ring-like LGE pattern) [8, 16, 90, 91]. Unless recurrent flares occur, oedema tends to decline 4 weeks after disease onset [88]. Myocardial LGE often appears to be less extensive in follow-up CMRs or may even disappear completely at 6 months (healed myocarditis) when it had been expression of oedema and not fibrosis [16]. In order to improve risk stratification and differentiation of convalescent myocarditis from healthy individuals, CMR in the follow may include parametric T1 and T2 mapping with calculated ECV values whenever possible.

## Future directions in myocarditis diagnostics

Although parametric mapping techniques have further increased the sensitivity of CMR to diagnose myocardial inflammation, the diagnostic accuracy in low-grade or chronic inflammatory disease might be hampered. Therefore, there is still a need for additional imaging markers to further improve diagnostic accuracy and risk stratification in patients with inflammatory myocardial disease.

CMR fingerprinting is a technique that allows for rapid and simultaneous acquisition of multiple, fully co-registered parametric maps within a single scan by matching complex signal measurements to a dictionary of simulated signals [92, 93]. It has the potential to improve diagnostic accuracy of myocardial maps by increasing the resolution and anatomic coverage, as well as substantially improving the reproducibility (enabling more reproducible measurements independent of center-specific hardware and patient physiology) [94, 95]. Moreover, it could extend myocardial tissue characterization beyond traditional mapping techniques by incorporating new parametric maps (e.g., diffusion or perfusion maps) and be used for comprehensive machine learning applications [96]. There are several other promising quantitative CMR techniques in preclinical evaluation that could help improve diagnosis of inflammatory myocardial disease in the future: cardiac diffusion-weighted imaging (cDWI) showed promising correlation between apparent diffusion coefficient (ADC) and LGE in chronic myocardial infarction [98]. In vivo studies of cardiac diffusion tensor imaging (cDTI) have shown its ability for microstructural and functional assessment of the myocardium, that might open up the road for detection of myocardial fiber remodeling also in inflammatory cardiomyopathy [97].

Artificial intelligence (AI) incorporated with machine learning and deep learning algorithms is going to revolutionize medical healthcare and in particular cardiovascular imaging. CMR lends itself to AI applications because it is based on complex image acquisition, reconstruction, segmentation/quantification, as well as image analysis and diagnostic reporting, which can be markedly improved by machine learning applications [98]. First AI applications for automated cardiac function analysis have already found their way into clinical use [99]. The aforementioned pre-clinical quantitative CMR techniques can benefit tremendously from machine learning algorithms. CMR fingerprinting directly profits from machine learning, as faster and more robust acquisition and reconstruction algorithms facilitate the generation of reproducible and unbiased maps needed for the development of machine learning applications [100]. Deep learning-based segmentation of LGE scars and parametric mapping could extend myocardial tissue characterization by improving reproducibility and sensitivity [101]. Furthermore, deep learning algorithms could decisively improve CMR techniques that are on the cusp of routine clinical application, such as functional strain analysis, by further improving its accuracy and reproducibility [102].

Machine learning approaches and big data analysis gave rise to another promising field, termed radiomics. Radiomics reflects a conversion of medical images into high-dimensional data and enables the extraction of various features (e.g., texture or filter features) that go beyond the conventional visual approach [98]. First radiomics and texture analysis applications in CMR using T2 mapping-derived texture features analysis showed superior diagnostic performance in patients with infarct-like and heart failure-like myocarditis [103, 104]. These novel texture analysis concepts could significantly improve the current challenges in diagnosis of low-grade or chronic myocardial inflammation or inflammatory cardiomyopathy (e.g., detection of subtle, diffuse or even visually non-assessable myocardial alterations).

**Fig. 7:**
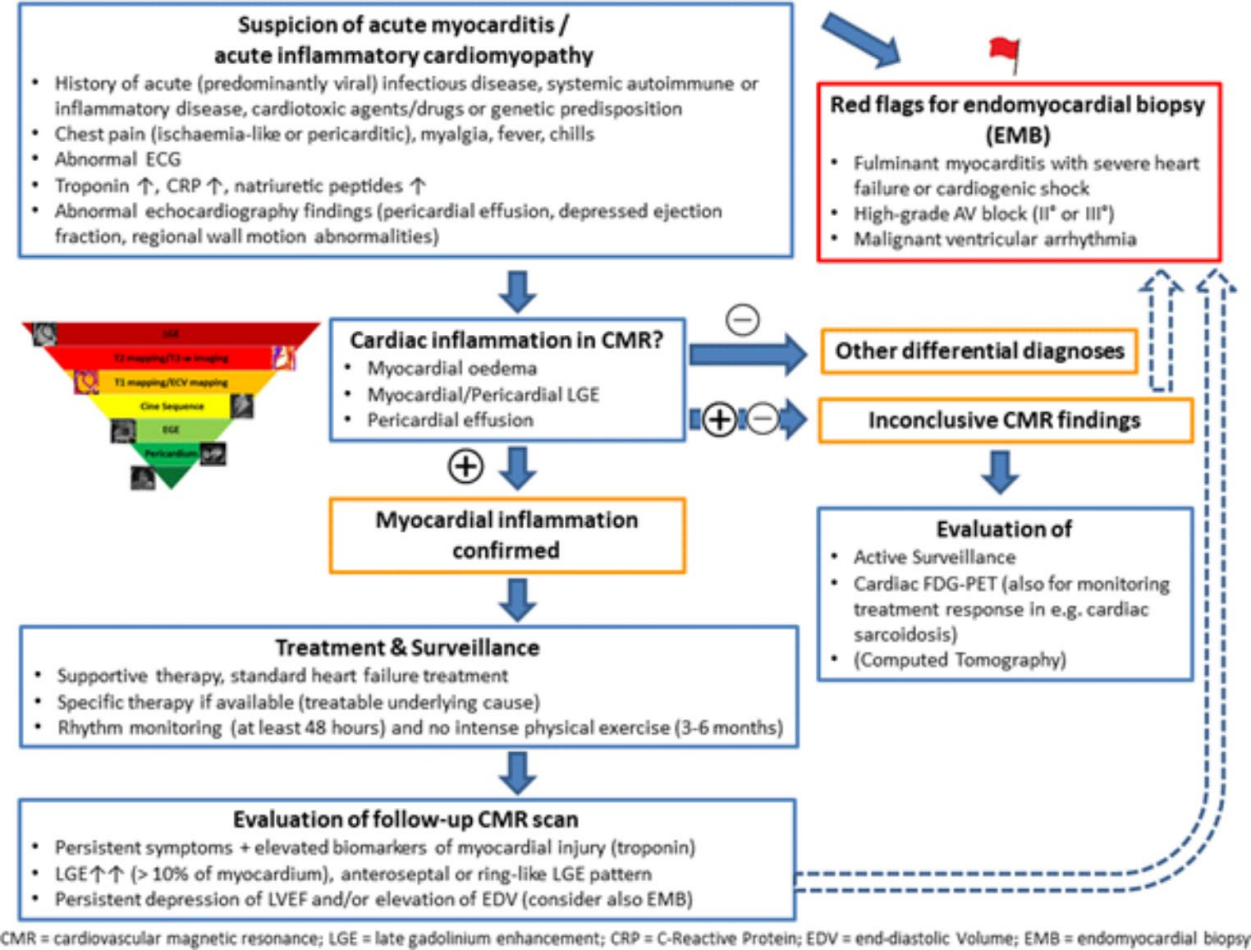
Clinical scenarios where acute myocarditis/acute myocardial inflammation may be suspected with a summary of guidance of diagnostic multi-modality imaging assessments, general treatment and surveillance recommendations. Clinical and CMR findings are proposed when a follow-up CMR scan should be evaluated

Furthermore, alternative imaging modalities such as spectral dual-energy and photon-counting CT could allow early detection of myocardial inflammation in routine clinical practice, where CT is typically performed before CMR. Hybrid imaging using PET, with its ability to detect focal and chronic inflammation, could be specifically incorporated into diagnostic algorithms for myocarditis and could further improve by the development of new tracers [50, 105, 106]. A proposal for a diagnostic algorithm is provided in Fig. 7.

## Conclusion

CMR represents an invaluable tool in the diagnostic work-up of acute myocarditis and chronic i-CMP. In some cases, adding ^18^ F-FDG may help in differentiating between acute and chronic i-CMP, thus allowing to choose the most effective therapeutic approach. Scarce data are available on hybrid PET/MR imaging, but combining the information coming from both morphologic and metabolic assessment may yield improved accuracy in selected cases, wherein the diagnosis is not clear.
